# Syphilization as Treatment for Syphilis

**Published:** 1868-08-01

**Authors:** N. Rosenberg

**Affiliations:** Chicago, Ill.


### SYPHILIZATION AS TREATMENT FOR SYPHILIS.

BY N. ROSENBERG, M.D., CHICAGO, ILL.

CASE No. 1.—Mr. D. C.; aged 40; native of Ireland; saloonkeeper by occupation; habits intemperate. Applied at my office June 1, 1866, and gave as history, that he had contracted syphilis 10 years previous, and had been treated by various physicians, and taken a large quantity of drugs, containing, as he believed, more or less mercury. When I first saw him, he presented the following symptoms: face flushed and swollen; complained of great weakness; lower extremities swollen, and on the anterior aspect of both were large, deep ulcers of several years standing, discharging fœtid matter in large quantity, so he was obliged to have a room for himself. I commenced to syphilize him, June 1st, 1866, after the manner prescribed by Prof. Boeck, and continued for four months, when further inoculation failed. At this time the sores on the legs were entirely healed, and his general health, according to his own statement, as good as it ever had been, and has subsequently remained so. He now holds a place as conductor on one of the street cars.

Case No. 2.—Mr. and Mrs. F. A.; natives of Germany; about 30 years of age; contracted syphilis in 1864; were treated for a long time in New York hospital, and after that by several physicians. Applied on the 8th day of May, 1866, presenting the following symptoms: the husband was very weak, had sore throat, and looked like a person suffering from consumption, and was unable to work, but had no sores on his body. The wife had several sores on different places, her eyes were affected, and she had a sickly appearance. Their child, two months old, died shortly before I knew them, of syphilis. I syphilized them the first time on the 8th of May, 1866. They left me, as completely cured, three months after. In September, 1867, they got a child, free from any symptoms of syphilis. The child is now ten months old, and is very healthy. It can be seen here in Chicago.

Case No. 3.—O. J.; 35 years of age; native of Norway; contracted syphilis in 1860; was in the American army during the war, and treated by several physicians while there. He states to have taken a great deal of mercury, but without any benefit. He had several boils and large discharging sores on many places. I commenced to inoculate him on the 12th of February, 1866. He went off as perfectly cured after treatment for three months and a half, and feels now better than he ever did, according to his own statement.

Case No. 4.—Mr. B. C. G.; 59 years of age; native of America; contracted syphilis in 1850; treated by several physicians; taken a great deal of mercury, and was given up by them as incurable. He had big discharging sores all over, could not wear boots, and could scarcely walk. I commenced to inoculate him on the 16th of May, 1866. The inoculation had a great effect upon him—the sores healed, and by and by he left me as completely cured, after four months treatment.

Case No. 5.—Mrs. L. McK.; 44 years of age; native of Ireland; contracted syphilis twelve years before I saw her; she confessed to have been a prostitute; had big sores on different places, and had been treated by many physicians. I commenced to inoculate her in 1865. She got perfectly well in three months.

Case No. 6.—Child, Miss O.; 10 years of age; native of Norway; supposed that she contracted syphilis in a privy, about four months before she came to me, at which time she was emaciated and anemic; she was rather poorly developed; extensive ulceration on both labia majores, with several smaller ulcers, and quite a number of condylomes about the rectal orifice; had had sore throat two months previous, but no eruptions on the skin; she had never been under treatment; her general health was poor, and she suffered great pain from the above mentioned difficulties. I inoculated her the first time on the 9th of July, 1866, and continued to treat her for a period of three months, when the sores were entirely healed, the condylones disappeared, and her general appearance so much improved, that she could hardly be recognized.

Case No. 7.—I. T.; 20 years of age; colored man; native of America; contracted syphilis five years before he came to me; had been treated by several physicians; he stated to have suffered much pain in the breast; had big discharging sores under both his feet; was very weak, and could scarcely walk. I commenced to inoculate him on the 30th of July, 1866, and he was perfectly cured after three months treatment, and he is now fatter and stouter than he ever was.

Case No. 8.—Mrs. C. L.; 45 years of age; native of Norway; contracted syphilis in August, 1867; was treated by an eminent physician here in this city for five months, but with no apparent benefit. I commenced to inoculate her on the 1st of March, 1868. She got completely cured in two months.

I have treated about three hundred syphilitic cases here in Chicago by syphilization. Many of those were given up as incurable. The treatment works here with astonishing effect, and I believe we shall, by syphilization, be able to cure all cases of secondary and tertiary syphilis.

